# Evaluating the provision of paediatric liaison psychiatry services in England

**DOI:** 10.1192/bjo.2022.638

**Published:** 2023-02-01

**Authors:** Declan Hines, Tamsin Ford, Sophie Westwood, Jessica R. Barrett, Birgit Westphal, Virginia Davies, William Lee

**Affiliations:** School of Clinical Medicine, University of Cambridge, UK; Department of Psychiatry, University of Cambridge, UK; Faculty of Health, University of Plymouth, UK; Department of Clinical Educational and Health Psychology, Centre for Outcomes Research and Effectiveness, University College London, UK; Paediatric Liaison Team, The Royal London Children's Hospital, UK; Paediatric Mental Health Team, Whittington Hospital, UK; School of Medicine, University of Exeter, UK

**Keywords:** Qualitative research, patients, in-patient treatment, epidemiology, clinical governance

## Abstract

**Background:**

Liaison psychiatry provision for children and young people in England is poorly evaluated.

**Aims:**

We sought to evaluate paediatric liaison psychiatry provision and develop recommendations to improve practice.

**Method:**

The liaison psychiatry surveys of England (LPSE) cross-sectional surveys engage all liaison psychiatry services in England. Services are systematically identified by contacting all acute hospitals with emergency departments in England. Questions are developed in consultation with NHS England and the Royal College of Psychiatrists’ Faculty of Liaison Psychiatry, and updated based on feedback. Responses are submitted by email, post or telephone**.** Questions on paediatric services were included from 2015 (LPSE-2), and we analysed data from this and the subsequent four surveys.

**Results:**

The number of acute hospitals with access to paediatric liaison psychiatry services increased from 29 (15.9%) in 2015 to 46 (26.6%) in 2019, compared with 100% provision for adults. For LPSE-4, only one site met the Core-24 criteria of 11 full-time equivalent mental health practitioners and 1.5 full-time equivalent consultants, and for LPSE-5, just two sites exceeded them. Acute hospitals with access to 24/7 paediatric liaison psychiatry services increased from 12 to 19% between LPSE-4 and LPSE-5. The proportion of paediatric liaison psychiatry services based offsite decreased from 30 to 24%.

**Conclusions:**

There is an unacceptable under-provision of paediatric liaison psychiatry services compared with provision for adults. Number of services, staffing levels and hours of operation have increased, but continued improvement is required, as few services meet the Core-24 criteria.

## The link between physical and mental health

Recent national goals for mental health research aim to reduce the excess mortality of those with severe and enduring mental health conditions, including halving the proportion of children with persistent mental health problems and improving our understanding of the interplay between mental and physical health.^1^

In paediatric populations, having a physical illness increases the odds of having a mental health condition by 82%.^2^ Further, emotional difficulties commonly complicate or even underpin physical health complaints treated in hospital settings. Recognising and treating these is a preventive medicine task that saves money via mechanisms that include better adherence to (often expensive) treatments, avoidance of unnecessary and potentially aversive investigations, and reducing emergency admissions and length of stay. Numerous studies have highlighted the high rates of psychiatric comorbidity in children with long-term physical health conditions,^2–5^ as well as the high cost of failing to address psychiatric comorbidity in this age group.^6^ Given that first episodes of mental illness occur before adolescence in 69% of individuals,^7^ and poor mental health in childhood and adolescence has been shown to affect subsequent health, social and occupational outcomes,^8^ there are strong clinical and financial arguments for early detection of mental illness in this population.

Given the strong evidence supporting the arguments for in-house paediatric mental healthcare, particularly in hospitals dealing with acute or severe illness, it is no surprise that there have been recent calls for greater integration of psychiatric and medical teams in these settings.^9^ Sadly, integrated mind–body care is often not available, and such integration is seldom included in service specifications. Instead, child and adolescent mental health services (CAMHS) are often asked to provide in-reach to their local hospital, predominantly for children and young people presenting in crisis, such as with self-harm. As a result of this arrangement, children and young people on wards and clinics are frequently unable to access any mental health input at all when in hospital, and opportunities for early detection of problems are missed.

## Targets for liaison psychiatry

Services that provide mental healthcare to patients in hospital are known as liaison services. They also have a less obvious but equally important role in detecting and managing the emotional difficulties described above. All acute hospitals should have access to a liaison service capable of supporting patients throughout their lifespan. Quality indicators for such services were developed by NHS England and include the 2016 ‘Core-24’ guidelines^10^ for adult liaison psychiatry services. These specify that services should operate 24 h a day, 7 days a week, and specify minimum staffing levels of 11 full-time equivalent (FTE) mental health practitioners (MHPs) and 1.5 FTE consultants. The UK Government set a target that at least half of England's general hospitals with operating emergency departments should have adult liaison psychiatry services meeting the Core-24 specification by 2020–2021.^11^ No such target has been set for child services, despite the expectation in the Five Year Forward View for Mental Health, published the same year,^11^ that all hospitals serving children have age-appropriate liaison services in place within the same time period.

## Rationale for analysing paediatric liaison psychiatry

Following the Care Quality Commission's statement in its 2020 report on ‘Assessment of Mental Health Services in Acute Trusts’^12^ that ‘acute trusts must do more’ for those with mental health problems, we analysed data from the liaison psychiatry surveys of England (LPSE), which are large surveys conducted approximately annually, describing liaison psychiatry services in England, and included paediatric services from 2015.^13–16^ The most recent was undertaken in 2019.

In exploring the identified services, and given the absence of NHS England quality indicators specifically designed for children's services, we drew on those developed by the Royal College of Psychiatrists’ Psychiatric Liaison Accreditation Network, who suggest that, as a minimum, the service must have procedures in place to refer children and young people to appropriate services, and identify patients on the child protection register; ensure all staff working with these patients have developmentally appropriate training; and ensure specialist clinicians and professional support are always available via an on-call system for children and young people.^17^

## Method

The LPSEs are national service evaluations commissioned by the Department of Health and Social Care, so did not require ethics approval.

### Sample

The sampling frame for the initial paediatric survey, which was conducted during LPSE-2, was developed by contacting the Child and Adolescent Faculty of the Royal College of Psychiatrists for a list of hospitals with a paediatric liaison service, and then including any additional hospitals known to those contacted initially (total 32 hospitals). Twenty-four hospitals responded and 19 confirmed the existence of paediatric liaison psychiatry, as well as reporting six more hospitals with paediatric liaison psychiatry services, and 15 without. None of the clinicians at the 24 contacted hospitals knew of any other services among the remaining 116 hospitals that provide acute medical services in England.

To ensure maximum reach, the procedure was modified for subsequent surveys by systematically contacting all acute hospitals with type 1 emergency departments listed with NHS England. Where services had already been contacted in LPSE-2, they were contacted directly again. Otherwise, initial contact was made with the adult liaison psychiatry services at each hospital to establish whether there was a paediatric service to include. The survey was also publicised via the Royal College of Psychiatrists’ paediatric liaison network email list, the adult liaison faculty email list and four psychology email lists, to reach the maximum number of liaison clinicians. As a result of this strategy, 177 hospitals were contacted for LPSE-3, 175 for LPSE-4 and 173 for LPSE-5.

### Measure

The areas covered in the bespoke questionnaire each year are described in Table 1. Amendments to the questionnaire between years were based on feedback and consultation with NHS England, the Royal College of Psychiatrists’ Faculty of Liaison Psychiatry and other stakeholders. A modified version of the adult LPSE-2 questionnaire was used in 2015, and subsequently, the same questionnaire was used for both adult and paediatric services. It should be noted that even the term ‘paediatric liaison psychiatry service’ is used variably across England. Some areas might view this as a single psychiatrist, and others as the CAMHS in-reach service rather than in-house mental health team members. This fluid terminology was managed by providing ‘free-text’ responses, so respondents could clearly describe their situation and the research team could then code it appropriately. The questionnaires are available in Supplementary Appendix 1 available at https://doi.org/10.1192/bjo.2022.638.

**Table 1:** Topic areas covered in each questionnaire showing amendments between years

LPSE-2	LPSE-3	LPSE-4	LPSE-5
Hospitals and Trusts served by the service	Hospitals and Trusts served by the service	Hospitals and Trusts served by the service	Hospitals and Trusts served by the service
Providers of the service	Providers of the service	Providers of the service	Providers of the service
Staff details (number of full-time equivalents, locums and fixed-term appointments)	Staff details (number of full-time equivalents, locums and fixed-term appointments)	Staff details (number of full-time equivalents, locums and fixed-term appointments)	Staff details (number of full-time equivalents, locums and fixed-term appointments)
Which patients the service sees	Which patients the service sees	Which patients the service sees	Which patients the service sees
Hours of operation	Hours of operation	Hours of operation	Hours of operation
	Competencies and training courses; outcome measures used by service	Competencies and training courses; outcome measures used by service	Competence framework and outcome measures used by service
Interaction of service with other services and mental health workers, including transfer between services	Interaction of service with other services and mental health workers, including transfer between services		
Resource levels relative to previous years	Resource levels relative to previous years	Resource levels relative to previous years	Resource levels relative to previous years
Response time standards	Response time standards	Target wait times	Target wait times
Research to develop service	Research to develop service		
	Other activities such as teaching and out-patient activities	Other activities such as teaching and out-patient activities	Other activities such as teaching and out-patient activities
		Challenges in the service	Challenges in the service
Budget			

LPSE, liaison psychiatry surveys of England.

### Procedure

Data collection took approximately thirteen weeks for each survey. All identified paediatric liaison psychiatry services were emailed a copy of the questionnaires. Emails were sent to respondents from previous years. Where there was no active email contact at a hospital, the acute hospital switchboard was telephoned to contact the hospital's liaison psychiatry services to request a suitable email address. Follow-up emails from local clinicians assisting the survey were sent to non-responders, and then telephone contact was made by the central survey team (W.L. and S.W.). Responses were submitted by email or post, whereas telephone responses were recorded by J.R.B. and W.L. for LPSE-3, and W.L. and S.W. for later surveys. This careful follow-up aimed to mitigate bias from non-response.

### Data management and analysis

Responses were entered into a secure Google sheet. Semi-structured data were manually coded with the aid of a coding protocol created in advance by W.L. and S.W., in consultation with the Royal College of Psychiatrists’ Faculty of Liaison Psychiatry (see Supplementary Appendix 2). The protocol was refined during the coding process. Using ‘free-text’ responses in this way aimed to avoid ‘forced-choice’ situations that might have led to misleading responses. The manuscript was written using the Standards for Quality Improvement Reporting Excellence reporting guidelines.^18^

## Results

**Fig. 1:**
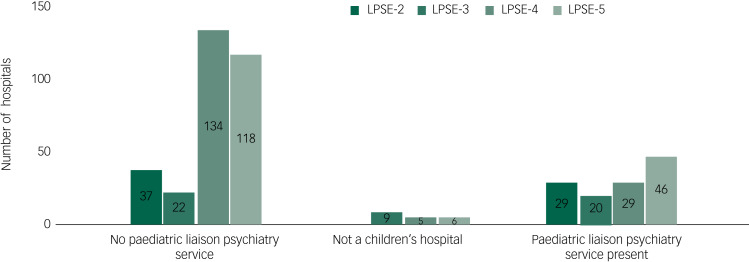
Number of acute hospitals with emergency departments with or without paediatric liaison services compared with responses to previous surveys, including cradle-to-grave services. LPSE, liaison psychiatry surveys of England.

There was a substantial increase in the number of hospitals with access to paediatric liaison psychiatry services between 2015 and 2019, both in raw numbers (from 29/182 to 46/173) and as a percentage of all identified acute hospitals (from 15.9 to 26.6%; see Fig. 1). Those hospitals with no paediatric liaison psychiatry service often expressed desire for one in the free-text answers. Differences between the number of identified hospitals each year and variation in the number of responses can be assumed to be a result of non-response by acute hospitals with no paediatric liaison psychiatry service, and this is higher in LPSE-2 and LPSE-3 because of differences in data collection methods (see above); hence why the number of hospitals with no paediatric liaison service is lower in LPSE-2 and LPSE-3. Also note that not every service answered every question.

**Fig. 2:**
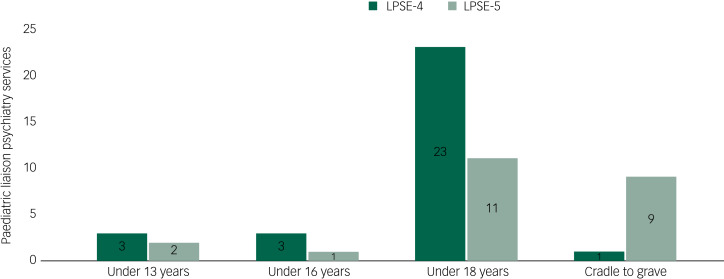
Age criteria for paediatric liaison psychiatry services, with results from LPSE-4 and LPSE-5. LPSE, liaison psychiatry surveys of England.

Responses from paediatric liaison psychiatry services included in LPSE-4 and LPSE-5 indicated an increase in the number of cradle-to-grave services, these being services under a single management covering the whole life course (from 3% in LPSE-4 to 36% in LPSE-5), and a corresponding decrease in services offered only to specific age ranges because these services were incorporated into the new cradle-to-grave services (see Fig. 2). Until 2019, there was also an increase in services reporting better resourcing compared with the previous year.

**Fig. 3:**
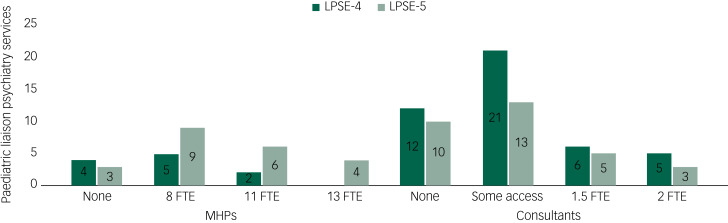
Mental health practitioner and consultant staffing levels in paediatric liaison psychiatry services, with results from LPSE-4 and LPSE-5. FTE, full-time equivalent; LPSE, liaison psychiatry surveys of England; MHP, mental health practitioner.

Importantly, there was only one acute hospital reporting to LPSE-4 about paediatric liaison that met the adult Core 24 criteria, whereas in LPSE-5, two hospitals exceeded them. Figure 3 shows an increase in staffing of services by FTE non-medical MHPs and a small decrease in no access to consultant child and adolescent psychiatry consultants between LPSE-4 and LPSE-5. Worryingly, FTE consultants and ‘access’ to consultants decreased, although reports of no access also decreased, making these figures difficult to interpret.

**Fig. 4:**
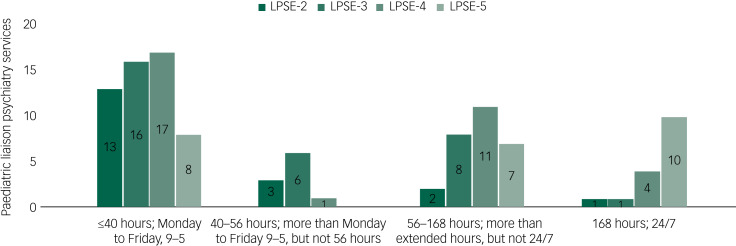
Hours of operation in paediatric liaison psychiatry services. LPSE, liaison psychiatry surveys of England.

There has been an increase in hours of operation (see Fig. 4). Notably, from LPSE-4 to LPSE-5, acute hospitals with access to 24/7 paediatric liaison psychiatry services increased from 12 to 19%.

The proportion of paediatric liaison psychiatry services based offsite decreased from 30 to 24% between 2018 and 2019. Paediatric liaison psychiatry services based on the same site as the acute hospital but in a different building to the emergency department have increased from 21 to 44%, whereas the number of paediatric liaison psychiatry services based in the same building as the emergency department declined from 45 to 28%. Community CAMHS was not counted as a paediatric liaison service, and so all services doing the emergency department work described in this section are in-patient liaison services.

## Discussion

These survey results suggest that, despite increases in provision, fewer than a third of acute hospitals provide universal access to age-appropriate paediatric liaison psychiatry services. Interestingly, these results are similar to a London-based service of paediatric liaison from 2001,^19^ and are in stark contrast to the now universal access to adult liaison psychiatry across England. As already noted, the well-evidenced arguments for co-located mental health provision are laid out in the Royal College of Paediatrics and Child Health ‘Facing the Future’ document.^20^ Despite these arguments, current levels of paediatric liaison psychiatry provision are inadequate to address the explicit, let alone the less obvious, mental health needs of children in hospitals in England, and this is without the increased demand in relation to COVID-19, particularly among children and young people with eating disorders.^21^

Some adult services are expanding to become ‘cradle-to-grave’ services, which could have obvious benefits for young people and emerging adults. However, training and qualifications for psychiatrists differs according to whether they work with children and young people, working-age adults or older adults. Although clinical psychologists all complete a training placement within CAMHS, most MHPs from other disciplines experience a generic training that rarely includes working with children and young people.^22^ Care must therefore be taken that these cradle-to-grave services employ suitably trained staff to work with children and young people.

Regardless of whether cradle-to-grave service staff have developmentally appropriate training, there is great variation in provision. Only nine out of 23 services report being cradle-to-grave, leaving the majority with service gaps for specific age groups, particularly those under 18 years of age. The free-text answers in LPSE-5 revealed further variability between services with similar age eligibility criteria, with some strictly enforcing the age cut-offs and even transferring patients between teams during a single episode of care, and others including factors such as social and educational context as determinants of eligibility. This particularly affects 16- and 17-year-olds, as those with the poorest mental and physical health are less likely to be in education or training than their healthier peers,^23^ and adult services rarely accept referrals for those under 18 years of age. This variability may well arise from the ongoing process of introduction of comprehensive paediatric liaison psychiatry services, which makes a repeat LPSE a priority to assess progress.

Adequate resources and central government targets are needed to achieve greater uniformity of age-eligibility criteria and, more generally, paediatric liaison provision would benefit from greater standardisation between services to ensure access and service quality is equitable across England. A centrally led effort is needed to expand both the number of acute hospitals with access to paediatric liaison services as well as to increase the number of such services reaching the Core-24 criteria. In addition, central direction is required to encourage appropriate mental health staffing levels in acute trust services for children and young people.^24^

The survey results suggest that, as well as being far fewer in number than their adult counterparts, paediatric liaison services are less accessible. Although there was an even split between services operating 9 a.m. to 5 p.m. Monday to Friday and those offering some extended hours, the decrease between LPSE-4 and LPSE-5 in 9–5 services and the consequent increase in 24/7 services is consistent with the general trend in adult liaison services.

The welcome expansion in crisis services to provide 24 h cover offers some support for children and young people, but is insufficient to support the efficient and effective management of children and young people in the emergency department or attending acute paediatric clinics or wards. Physical long-term conditions place many at higher risk of poor mental health, whereas some mental health conditions, such as eating disorders, undermine physical health and require integrated care packages. It is also essential to provide sufficient staff to manage the emergency demand without compromising the offer to non-acute patients.

The interface between crisis and liaison services varies depending on local service organisation and relationships between trusts. Where providers secured ‘crisis money’ to fund extended crisis teams, the interface to liaison psychiatry relies on service-level agreements, which are clearly easier to develop if both services are provided by same trust. Where liaison psychiatry is provided by the acute trust, the interface to extended crisis service or community CAMHS can be more challenging, particularly in the absence of clear collaborative protocols. We need national guidance for integrated care services to standardise and optimise joint working to avoid duplication or gaps in provision. Future research should examine variability in provision across the country to provide a template for best practice, given different service constellations.

### Limitations

LPSE is the most thorough survey of staffing of paediatric liaison services, with strenuous efforts made to establish a true denominator and increase response rates. However, in part because of increased responding, it is difficult to know how much the apparent growth in paediatric liaison psychiatry provision in England captured by our survey results is real and how much is artefactual. The relatively small numbers of services involved means that small changes may appear disproportionately large. The surveys also cover a period, especially between LPSE-4 and LPSE-5, when local health and care bodies (initially involved in sustainability and transformation programmes and now becoming integrated care systems) were investing heavily in meeting the government's demand that under 18 s be able to access 24/7 emergency mental healthcare. This resulted in the creation of the large crisis teams discussed above, covering geographically larger patches than one acute NHS trust. It is therefore possible that some respondents were reporting increasing staffing levels because of increased crisis provision, rather than a growth in true in-house paediatric liaison services.

The questionnaires were necessarily bespoke, and it is possible that respondents misunderstood the definition of a liaison service; for example, including crisis services serving the community, but sometimes based in acute hospitals, instead of in-house mental health services serving patients of the hospital. Similarly, comparison across years is limited by changes in the questionnaire, but these variations aimed to improve the utility of findings and were deemed necessary, based on stakeholder consultations. Finally, we lacked standards for paediatric liaison and so applied those for adult services, as most seem transferable. The least easy to translate is the staffing requirements; however, given that two of the most common presentations to liaison services are self-harm and eating disorders, with a peak age of presentations in the mid to late teens, this seems reasonable in the absence of other clear data.

### Future recommendations

Central direction is urgently needed to improve the provision of liaison psychiatry to children and young people. Future research should address the limitations in our results and capture the demand for out-of-hours services by age group, to assist in targeting funding and service provision according to need. While keeping the questions as consistent as possible from previous surveys and across services serving all age groups, we should ensure that key issues specific to children and young people are covered. Triangulation against other data sources, such as administrative data, would help to verify the responses. Further work should also identify whether every district general hospital has a lead for paediatric mental health, in accordance with Royal College of Paediatrics and Child Health guidance, and should explore the interface between paediatric psychology and liaison services, as the former were largely not included in these surveys of liaison psychiatry services. Urgent funding of regular LPSEs is vital to document progress in service provision or indeed decommissioning, particularly given the recent increases in demand.^23^

## Data Availability

Data are available from the corresponding author upon reasonable request.

## Supplementary material

For supplementary material accompanying this paper visit http://doi.org/10.1192/bjo.2022.638. click here to view supplementary material
