# Supplementary material for: Evaluating the provision of paediatric liaison psychiatry services in England
Source: BJPsych Open. 2023 Feb 1;9(2):e30. doi: 10.1192/bjo.2022.638 (PMC9970163; doi:10.1192/bjo.2022.638)
Supplement: Supplementary file 1 [file bjosup.zip › S205647242200638Xsup001.docx]

**LPSE 2 – Questions asked of working age adult and older services**

| 1 | What is the name of your Liaison Psychiatry service (if it has one)? |
| --- | --- |
| 2 | What is the name of the Acute Hospital(s) you are based in? |
| 3 | What is the name of the Acute Trust(s) you are based in? |
| 4 | Does the Acute Trust(s) have more than one site with inpatient beds?  If so, please name them. |
| 5 | Does the Acute Trust(s) have more than one A&E? if so, please name them. |
| 6 | Does your Liaison Psych service provide services to all the sites? |
| 7 | If not, can you give us a contact details of the other liaison psychiatry service(s) please? |
| 8 | What is the provider of your service?  (Usually this is the mental health trust) |
| 9 | Is psych liaison in your Acute Trust provided by one or many providers?  If many, which? |
| 10 | If the above questions do not capture details of your service, please explain. |
| 11 | What services do you provide, and to whom?  (Some only see self-harms, some see anyone in the whole hospital, others are in-between. Some look after alcohol problems, some not, some do LD, some not, etc.)  What are the age-criteria for your service(s)? |
| 12 | Do you support anything other than the acute care pathway?  Are there any clinics, etc.  If so, can you outline the nature of the work? |
| 13 | Number of FTE nurses and their bands? |
| 14 | Number of FTE doctors and their grades? |
| 15 | Number of FTE admins and their grades? |
| 16 | Number of other clinicians and their grades if known? |
| 17 | Number of other non-clinicians and their grades if known? |
| 18 | Of the above, who is substantive and who is a locum, part of winter pressures. fixed term appointments, etc? |
| 19 | What is your service’s budget, if known?  (Leave out the medics (or just junior medics) if necessary). |
| 20 | How much of that that budget is permanent and how much is temporary  (if known)? |
| 21 | What are your service’s hours of operation?  (Out Of Hours SHO cover does not mean your service is 24/7). |
| 22 | Does your service do all the work contained in all the referrals? (eg is some passed on to other services? Please explain.  (This question is about things like requests for psych opinions from wards, which are sometimes passed straight on to the duty SHO) |
| 23 | Are there other mental health workers in your acute trust who are not part of your service?(eg counsellors, psychologists) |
| 24 | Have you undertaken any research (published or not) to support the development of your service?  If so, can you describe it please? |
| 25 | Is your service better resourced than it was a year ago?  If so, how?  If worse, please also explain. |
| 26 | If the services are separate, how do people transfer from CAMHS to Working Age Adults and from Working Age Adults to Older Persons?  (This is usually age cut-offs plus exceptions and complications. There seems to be huge variety in this and we would like to catalogue it.) |
| 27 | Does your service have a response time standard and is that time agreed with referrers and/or commissioners? |

*Note: FTE = Full Time Equivalent.*

**LPSE 2 – Questions asked of services for children and young people**

| 1 | What is the name of your Paediatric Liaison Psychiatry service (if it has one)? |
| --- | --- |
| 2 | What is the name of the Acute Hospital(s) you are based in, and where is your team located within the hospital? |
| 3 | What is the name of the Acute Trust(s) you are based in (if relevant)? |
| 4 | a) Does your Paediatric Liaison service provide services to more than one site with an A&E? b) What other sites does your Paediatric Liaison service provide services to (if any)? |
| 5 | What is the provider of your service? Usually this is the mental health trust |
| 6 | Is Paediatric Liaison in your Acute Trust provided by one or many providers? If many, which? |
| 7 | Who commissions your service? |
| 8 | To capture the size of your Paediatric department, could you please say: How many Paediatric beds there are within the hospital? How many Consultant Paediatricians there are within the hospital? |
| 9 | To help us capture all Paediatric Liaison services.. …please name any acute hospitals which you know **do** have a Paediatric Liaison service, and… …please name any acute hospitals which you know **do NOT** have a Paediatric Liaison service. |
| 10 | What are the age-criteria for your service(s)? (We are aware this can be very complicated but we want to know all the details please, including whether it is different for known vs unknown patients, criteria about full time education, etc) |
| 11 | How are referrals processed, and is this different when urgent and non-urgent? |
| 12 | What services do you provide, and to whom? eg Self-Harm, Other ED presentations, Inreach to hospital wards (ward work), Short term follow up clinics (after people on caseload have been discharged),Clinics with other specialties which take outpatient referrals, Standalone clinics which take outpatient referrals Substance misuse, any additional services. |
| 13 | Do you take part in any MDTs or do any other joint clinical work? |
| 14 | Do you provide any staff support or any staff training groups? |
| 15 | Number of trained nurses and their bands? (Please give both the number of ‘bodies’ and the number of FTE posts) |
| 16 | Number of doctors and their grades (including training grades for juniors)? (Please give both the number of ‘bodies’ and the number of FTE posts) |
| 17 | Number of psychologists and their bands? (Please give both the number of ‘bodies’ and the number of FTE posts) |
| 18 | Number of other clinicians and their bands? (Please give both the number of ‘bodies’ and the number of FTE posts) |
| 19 | Number of admins and their bands? (Please give both the number of ‘bodies’ and the number of FTE posts) |
| 20 | Number of other non-clinicians and their bands? (Please give both the number of ‘bodies’ and the number of FTE posts) |
| 21 | Of the above, who is substantive and who is locum, part of winter pressures, fixed term, etc.? |
| 22 | Are there any other paediatric mental health workers in your acute trust who are not part of your service? (e.g. counsellors) |
| 23 | What is your service’s budget, if known? (Leave out the medics, or just junior medics, if necessary) |
| 24 | How much of that budget is permanent and how much is temporary (if known)? |
| 25 | What are your service’s hours of operation? |
| 26 | What happens outside of these hours? (Is there an on call SHO, or another service?) |
| 27 | Does your service do all the work contained in all the referrals or is some passed on to other services? Please explain. (This question is about things like requests for psych opinions from wards, which are sometimes passed straight onto the duty SHO) |
| 28 | Have you undertaken any research (published or not) to support the development of your service? If so, can you describe it please? |
| 29 | Is your service better resourced than it was a year ago? If so, how? If worse, please also explain. |
| 30 | Does your service have a response time standard and is that time agreed with referrers and/or commissioners? |

**LPSE 3 – Questionnaire (Adult and Childrens' services shared questionnaire from this point on)**

Third Annual Survey of Liaison Psychiatry in England (LPSE-3)

**Introduction:**

The questions in this survey are directed to Liaison Psychiatry services within acute hospitals in England. This survey is a lot more thorough than those which have gone before and therefore is more complex to fill out. However, we have worked to keep it simple, relevant and not too long. There are seven sections, each of which have a set of related questions about your service.

There is usually more than one service providing Liaison Psychiatry within one acute hospital (eg separate Older Adults, Paediatrics, etc.) and we ask for one response per service, please.

Before you start, you may want to check someone from your service has not responded already by looking at this link:

<https://docs.google.com/spreadsheets/d/1BKRZWfZdRtyR_C31JNe3PE42pjb2hame6L1i2VAhyM0/edit?usp=sharing>

Please reply to this email ([jessica.barrett@plymouth.ac.uk](mailto:jessica.barrett@plymouth.ac.uk)) and fill in the answers after each question in the included text. We’ll take it from there. If you’d rather do it by phone, please get in touch and we’ll arrange that too.

Thank-you very much for taking part in the 3rd Annual Survey of Liaison Psychiatry in England (LPSE-3)

**Questionnaire:**

1. Service overview. (7 questions)

1. What is the name of the service or team which provides Liaison Mental Health services on behalf of which you are responding?
   (We will call this ‘your service’ throughout)
2. Does your service offer anything other than Liaison?
   If so, please outline the other services.
   (Some teams are unified Crisis, Home Treatment and Liaison, for example.)
   (Many paediatric services offer Liaison as one of many activities undertaken.)
3. Is your service physically based in an acute hospital building?
   If not, please say how far away it is from the acute hospital.
   If so, what is/are the names of the acute hospital(s) site(s) in which your service is based?
4. Does your service provide Liaison to any other site(s)?
   If so, please name them.
   (This may be another acute hospital, GP surgery, or something else)
5. What is the name of the provider of your service?
   (Usually this is a Mental Health Trust).
6. Are there any funding anomalies?
   (eg Some services are paid for by only one CCG but see patients from any CCG, which can cause friction with commissioners. Please give details.)
7. What other mental health care is provided for people in your acute hospital(s) that you know about, and how does your service fit into that context?
   Please list those you know about and give contact details if you have them.
   (This question is about two things. It is about other Liaison services (eg paediatric, working age adult, older adult, self-harm, ED, ward referrals, Liaison for people who live in a different area, etc). It also about other mental health practitioners working in acute hospitals (eg counsellors/psychologists/health psychologists). There is particular policy interest in the relationships between paediatric Liaison Psychiatry and other paediatric mental health workers in acute hospitals.)

2. Workforce. (5 questions)

For each of the following questions, a-e, for each professional group please list the number of Full Time Equivalents at each level of seniority; The number of actual different people at each level of seniority; Please also detail who is substantive, who is a locum, who is temporary/fixed term, who is part of winter pressures, etc.

NB: If your service delivers care other than Liaison, please only include workforce figures for the Liaison part if you can. If there is no clear division, please describe the entire service and indicate approximately what fraction of the workload is Liaison.

a. Nurses - please include bands and details of advanced practitioners, prescribers, etc.

I. Band 5s

II. Band 6s

III. Band 7s

IV. Band 8s

b. Doctors - please include grades of juniors and include special interest sessions etc.

I. F1s

II. F2s

III. CT1-3s (=SHOs)

IV. ST4-6s (=SpRs)

V. SASs (=Staff grade/Associate specialists)

VI. Consultants

c. Psychologists (and bands if known)

d. Admins (and bands if known)

e. Other clinicians/non-clinicians (and bands if known)
 *(e.g. social workers, housing support officers, peer support workers…)*

3. Competencies. (5 questions)

a. Does your service use a competence framework? Please give details.

*(eg Liaison Mental Health Nursing Competence Framework).*

If yes, please specify which framework and for which staff groups these are used, as well as how they are used. If no competence framework is actually in organisational use, but its existence is known and there are plans to use it, please document this too.

b. Does your service use any accredited training courses for psychiatric liaison nurses?

(e.g. City University certificate in Liaison Mental Health)

c. What CCTs do the consultants in your team hold? Are these CCTs endorsed in Liaison Psychiatry and/or Addictions Psychiatry?
(Consultants all have a ‘Certificate of Completion of Training’ (CCT). These may be ‘endorsed’, which means the consultant had special experience and training in certain specialities as a junior doctor. Consultants’ CCTs are likely to be in General Adult Psychiatry, Old Age Psychiatry, Child and Adolescent Psychiatry, or a combination of these. Endorsements can be in Liaison and/or Addictions.)

d. Are members of your team routinely expected to see patients for whom they have not had special age-specific training?
(eg Older adults/Children and Young People)

e. Does your service have an induction/training package for new starters? If so, please give some details of length/content/provenance.

4. Activities undertaken by your service. (14 questions)

a. Referrals from the Emergency Department.

I. Do you accept and see absolutely all referrals from the ED, with no exclusions or criteria?
(This is not about people who are referred in error. It is about people who do need to be seen but do not meet your service’s criteria)
If not, what are the exclusions/criteria?
(Please be as specific as you can, particularly over timings: There is policy level interest in things like detoxes being overseen by alcohol services in hours but by Liaison out of hours. Similarly, if there is a difference between what is agreed and what actually happens, please document this too.)

II. What are the hours of operation of your service’s ED activities?

III. What happens outside these hours?
(Who sees the patients? PLN? SHO? HTT? Crisis? Just ED?)
(Who provides senior cover? eg Liaison consultant? Other consultant?)

IV. Do you have a target wait time or similar to see people referred from ED? If so, please give details.

V. Please give names (and contact details if you didn’t give them in Section 1) for all the other services which may see the people with mental health needs in ED whom your service does not see.
(eg Children, the elderly, substance users, people with dementia, people from out of area, people with learning disabilities, women in the perinatal period, etc.)

b. Referrals from wards (including MAU and CDU) in the acute hospital.

I. Do you accept and see absolutely all referrals from wards in the acute hospital, with no exclusions or criteria?
If not, what are the exclusions/criteria?

II. What are the hours of operation of your services’ ward referral activities?

III. What happens outside these hours?
(Who sees the patients?)
(Who provides senior cover?)

IV. Do you have a target wait time or similar to see people referred from wards in the acute hospital? If so, please give details.

V. Please give names (and contact details if you didn’t give them in Section 1) for all the other services in your acute hospital which may see people referred from the acute hospital wards whom your service does not see.

c. Outpatient activity.

I. Short term follow-up of people who have been seen by your service as an inpatient or ED attendee.
(We know of services which follow up people who have had delirium and services which follow up people who have harmed themselves.)

II. Clinics to which patients are referred, either by other specialist teams, from primary care, from out of area, or elsewhere.
(These clinics could be ‘general’ psychological medicine clinics, more specialist clinics for specific problems like MUS or chronic pain, clinics specifically related to particular specialities (eg diabetes). Patients may be seen by a Liaison professional alone or with a clinician from the other service. We know of existence of clinics for Medically Unexplained Symptoms, Neuropsychiatry, Pain, STD, Oncology, Neurology, Gastroenterology, Dermatology, Cystic Fibrosis, and Perinatal Medicine.)

d. Teaching.

Many Liaison services deliver teaching to ED staff, ward staff, managers, students, postgraduates etc.
Please describe all that happens, with an approx. total number of hours/year delivered. Please also document any student attachments with your service.

e. Other work. Please record any work undertaken by your service not captured above.

5. Interfaces with other teams: (10 questions)

a. Is there a Children and Young People’s Liaison Mental Health service, separate to your service, within the acute hospital?
If so, do you have any formal links with that service?

b. Is there a Learning Disabilities service, separate to your service, within the acute hospital?
If so, do you have any formal links with that service?

c. Is there a Working Age Adult Liaison Psychiatry service, separate to your service, within the acute hospital?
If so, do you have any formal links with that service?

d. Is there an Older Person’s Mental Health/Dementia/Frail and elderly Liaison Psychiatry service, separate to your service, within the acute hospital?
If so, do you have any formal links with that service?

e. Is there an Addictions/Substance Misuse service, separate to your service, within the acute hospital?
If so, do you have any formal links with that service?

f. Specialist clinical links to named teams:
Many services provide specialist expertise to particular named teams or departments. Here, there are organisational links beyond the sending of referrals. Examples might include a presence in an MDT or outpatient clinic of another service. Please outline the nature of the link and whether the extra resources are specifically commissioned or not: *(Examples of specialities with professional links include: Addictions, Bariatric , Cancer, Diabetes, Endocrinology, Gastroenterology, Gerontology, Hematology, Hepatology, Infectious Diseases, Perinatal/Obstetrics/Gynaecology, Medicine, Neurology, Neurosurgery, Orthopedics, Other (please specify), Pain Management, Palliative, Pediatrics, Rehabilitation, Renal, Respiratory, Spinal, Stroke, Toxicology, Transplant, Trauma)*

g. Is your service permitted to refer people with memory problems directly to your local memory assessment services?
(In many places these requests have to go via the GP. This is about direct referrals.)
(If your service does not see people with memory problems, please document this).

h. Please only answer these questions if your service sees Children and Young People:
I. How many Paediatric beds are there in the acute hospital?
II. How many Consultant Paediatricians are there within the hospital?
III. Do you have any intensive care/neonatal beds? If so how many?

6. Outcome measures used by your service. (4 questions)

a. Do you use FROM-LP in any of your clinical activities?
If so, please indicate whether you use the tool routinely or occasionally.

b. Do you use any other outcome measures? (eg the GOMS).
If so, please indicate whether you use the tools routinely or occasionally.

c. If you do use FROM-LP, please indicate which elements are used:
I. IRAC
II. CGI-I
III. CORE-10
IV. Patient Satisfaction Scale
V. Friends & Family Test
VI. Referrer Satisfaction Scale.

d. How might FROM-LP be improved?
(eg to make it easier to use or more useful)

7. Roundup questions: (7 questions)

a. Please list your service’s Key Performance Indicators, if you have any.

b. Has there been any research or audit (published or not) to support the development of your service?
If so, can you describe it please?

c. Is your service worse/similarly/better resourced than it was in April 2015?

d. Is there is an expectation of changes in service configuration or staffing in the next year? or five years?

e. What does your service do well?

f. What in your service is a challenge?

g. What help do you need from NHSE, HEE, the College, and generally, to provide a better service?

THANK YOU FOR TAKING PART IN THE THIRD ANNUAL SURVEY OF LIAISON PSYCHIATRY IN ENGLAND (LPSE-3)

**LPSE 4 – Questionnaire**

**Service overview (6 questions)**

**1. What is the name of the service or team which provides Liaison Psychiatry on behalf of which you are responding?** *(We will call this ‘your service’ throughout.) (If your hospital has more than one liaison service, more than one of these questionnaires should be completed. However, we will collate the results from each acute hospital for adults’ and older adults’ services, so whether there is a response for each of these, or one across both, does not matter as long as everyone is counted. Do what is easiest for you.)*

**2. Does your service offer anything other than Liaison? If so, please outline the other services.** *(Some teams are unified Crisis, Home Treatment and Liaison, for example.) (Many paediatric services offer Liaison as one of many activities undertaken.)*

**3. Is your service physically based in an acute hospital building?** *If not, how far away it is from the acute hospital? If so, what is the name of the acute hospital site where your service is based?*

**4. Does your service provide Liaison to any other sites?** *If so, please name them. (This may be another acute hospital, GP surgery, or something else)*

**5. What is the name of the provider of your service?** (*Usually this is a Mental Health Trust).*

**6.** **Is your service securely and recurrently funded?** I*s the whole service funded recurrently? If the term is fixed but long, please tell us when it is for review. Please describe if some or all of your service is recurrent but some is on short-term contracts. (There is policy interest in financial stability/instability of services, particularly ones which need to rejustify their existence regularly.)*

**Workforce (4 questions)** *For each of the following questions, for each professional group please list: The number of Full Time Equivalents at each level of seniority; The number of actual different people at each level of seniority; Please also detail who is substantive, who is a locum, who is temporary/fixed term, etc. If your service delivers care other than Liaison, please only include workforce figures for the Liaison part if you can. If there is no clear division, please describe the entire service and indicate approximately what fraction of the workload is Liaison.*

**7. Mental Health Practitioners** *(=Largely nurses) Please include bands and, if applicable, please identify which practitioners are primarily orientated in their role towards one client group or another (eg Older people/Children, etc*

Band 5s
Band 6s
Band 7s
Band 8s

**8. Doctors** *Please include grades of juniors and include special interest sessions etc.*

F1s

F2s

CT1-3s (=SHOs)

ST4-6s (=SpRs)

SASs (=Staff Grade/Associate specialists)

Consultants

**9. Others** *(and bands if known) e.g. Psychologists, psychotherapists, social workers, housing support officers, healthcare assistants, pharmacists, drug and alcohol workers, etc.*

**10. Administrators** *(and bands if known)*

**Competencies (2 questions)**

**11. Does your service use a competence framework?** *Please give details, particularly in how one is used. (eg Liaison Mental Health Nursing Competence Framework). If no competence framework is actually in organisational use, but its existence is known and there are plans to use it, please document this too.*

**12. What CCTs do the consultants in your team hold?** *(Consultants all have a ‘Certificate of Completion of Training’ (CCT). These may be ‘endorsed’, which means the consultant had special experience and training in certain specialities as a junior doctor.) (Consultants’ CCTs are likely to be in General Adult Psychiatry, Old Age Psychiatry, Child and Adolescent Psychiatry, or a combination of these. Endorsements can be in Liaison and/or Addictions.)*

**Activities (12 questions)**

**13. What are the criteria by which your service would accept a referral from the ED?** *Please be as specific as you can. (The most usual criterion is one of age, but there are many others, and they often change over time as well. There is great policy level interest in things like this, so please be really detailed.)*

**14. What teams or services would see the ED referrals who need to be seen, but your service does not see?** *Please be as specific as you can. (eg “The children (0-17) are seen by CAMHS or are admitted to paeds. The older people are seen by our older adults service from M-F 8am-7pm, but out of those hours those referrals come to us. People with primary alcohol or drugs presentations are seen by A&Es own alcohol worker in hours, but out of hours come to us.”)*

**15.** **What are the hours of operation of your service’s ED activities?** *(This is about the functioning of your service. Transferring over to the on call SHO out of hours, or a crisis team, does not make a service 24 hours)*

**16. What happens outside these hours?** *(Who sees the patients? PLN? SHO? HTT? Crisis? Just ED? They wait?)*

**17. What is the target wait time to see people referred from ED, if any?**

**18. What are the criteria by which your service would accept a referral from the wards (including MAU)?** *Please be as specific as you can.*

**19. What teams or services would see the Ward/MAU referrals who need to be seen, but your service does not see?** *Please be as specific as you can.*

**20. What are the hours of operation of your services’ ward referral activities?**

**21. What happens outside these hours?**

**22.** **What is the target wait time to see people referred from wards, if any?**

**23. What are the criteria by which your service would see outpatients?** *(If your service sees any outpatients at all. If there is more than one pathway into being seen as an outpatient, please list them all and their criteria.)*

**24. What other activities does your service undertake, if any?** *(Many Liaison services deliver teaching to ED staff, ward staff, managers, students, postgraduates etc. Please describe all that happens, and how frequently. Please also document any student attachments with your service. Please record any work undertaken by your service not captured above, in particular there are reports of non-ED s136s being undertaken by some Liaison services. This is of policy interest, so please include it here.)*

**Final queries (4 questions)**

**25. Which outcome measures do you use, if any?**

**26. Do you use the FROM-LP outcome measure?** *If so, please indicate which elements are used:*

IRAC

CGI-I

CORE-10

Patient Satisfaction Scale

Friends & Family Test

Referrer Satisfaction Scale

**27. Is your service worse, similarly, or better resourced than it was in July 2016?**

**28. What does your service do well?**

**29. What in your service is a challenge?** (In particular, are certain groups disadvantaged, especially age groups? *There is policy interest in whether unifying working age adult and older adult teams has resulted in older people being disadvantaged because of dilution of expertise and management being more focused on the needs of adults of working age.) (Everything you write is in confidence.)*

**30. Is there anything else you would like to contribute to the survey?**

**THANK YOU FOR TAKING PART IN THE FOURTH SURVEY OF LIAISON PSYCHIATRY IN ENGLAND (LPSE-4)**

**LPSE 5 – Questionnaire**

This is the 5th Liaison Psychiatry Survey of England. We are interested in the staffing and activities of services which provide mental healthcare in Acute Hospitals with Emergency Departments in England. All responses are read and understood by a human, so please just describe your situation if the questions don’t describe your service/situation very well.

***Your details:***

**1. Your name and contact details (email and/or telephone):**

***Service overview:***

**2. Name of acute hospital and trust in which your team functions:** *(Most Liaison Psychiatry services operate in a single acute hospital. If that is not your situation, please describe it).*

**3. What is the name of the provider of your service?** *(Usually this is a Mental Health Trust*

**4. What is the name of your team?** *(eg Working Age Adult Liaison Psychiatry, Older Adult Liaison Psychiatry, Dementia Team, Drug and Alcohol, Children and Young People, HIV, Adult all age Liaison Psychiatry)*

**5. Does your team offer Liaison Psychiatry to any other sites?** *(This may be another hospital, GP surgeries or something else)*

**6. Are there any other Liaison Psychiatry teams operating in your acute hospital? If so, please name and provide a contact for these other teams:** *(We want every liaison professional to be counted once. You can do this by each service responding separately, or by including the people in all the services in a single response, or some combination)*

**7. Is your team’s office in the same building as ED/wards? If not, is it on site? If not, what is the travel time (and mode of transport)?**

**8. Does your service/team offer anything clinical other than Liaison? If so, please outline the other clinical activities:** *(Some teams are unified Crisis, Home Treatment and Liaison, for example. Many paediatric services offer Liaison as one of many activities undertaken.)*

**9. Is your service securely and recurrently funded?** *(If the term is fixed but long. Please tell us when it is up for review. Please describe if some or all of your service is recurrent but some is on short-term contracts. Please say if your service has to rejustify its existence at intervals or anything like that).*

***Workforce:***

*(If your service delivers care other than Liaison, please only include workforce figures for the Liaison part if you can. If there is no clear division, please describe the entire service and indicate approximately what fraction of the workload is Liaison.)*

**10. MHPs and Doctors:**

| **Role description & Band/Grade** | **No. of People** | **No. of FTEs** *(Please only include time assigned to this service)* | **Employment Status** *(Substantive? Locum? Temporary? Fixed term? Winter pressures?)* |
| --- | --- | --- | --- |
| **Administrators Band 2** |  |  |  |
| **Administrators Band 3** |  |  |  |
| **Administrators Band 4** |  |  |  |
| **Administrators Band 5** |  |  |  |
| **MHP Band 5** |  |  |  |
| **MHP Band 7** |  |  |  |
| **MHP Band 7** |  |  |  |
| **MHP Band 8** |  |  |  |
| **Dr F1** |  |  |  |
| **Dr F2** |  |  |  |
| **Dr CT1-3s (=SHOs)** |  |  |  |
| **Dr ST4-6s (=SpRs)** |  |  |  |
| **Dr SASs (=Staff Grade/Associate specialists)** |  |  |  |
| **Others with non-MHP roles, and their bands:** *(eg. Associate Physician, Social Worker, Pharmacist, Drug and Alcohol Worker etc.)* |  |  |  |

***11. Consultants:*** *Please use one row for each consultant.*

| **FTE** *(Please only include time assigned to this service)* | **Certificate of Completion of Training (CCT)** *(General Adult, Old Age, CAMHS, etc.)* | **Endorsements** *(Liaison, Addicitions, etc.)* | **Employment status** *(Substantive? Locum? Temporary? Fixed term?)* |
| --- | --- | --- | --- |
|  |  |  |  |
|  |  |  |  |
|  |  |  |  |

***Activities:***

**ED**

**12. Service referral criteria (incl. age range) from ED:** *(Please be as specific as you can, and please specify here whether you always/sometimes/never wait for patients to be ‘medically fit for discharge’, or ‘medically cleared’ before seeing them.)*

**13. Which team/s or service/s see ED referrals you do not?** *(Please be as specific as you can: Tell us who sees patients in different age ranges, out of hours, primary alcohol presentations etc. or if they are not seen)*

**14. Hours of service (incl. number of days per week):** *(Transferring over to the on call SHO out of hours, or a crisis team does not count.)*

**15. What happens outside the above hours?** *(Who sees the patients? Do they wait until next shift?)*

**16. What are the target wait times to see ED referrals (if any)?**

**WARDS**

**17. Service referral criteria (incl. age range) from wards:** *(Please be as specific as you can, and please specify here whether you always/sometimes/never wait for patients to be ‘medically fit for discharge’, or ‘medically cleared’ before seeing them.)*

**18. Which team/s or service/s accept ward (incl. MAU) referrals you do not? (***Please be as specific as you can: Tell us who sees patients in different age ranges, out of hours, primary alcohol presentations etc. or if they are not seen)*

**19. Hours of service (incl. number of days per week):** *Transferring over to the on call SHO out of hours, or a crisis team does not count.*

**20. What happens outside the above hours?** *(Who sees the patients? Do they wait until next shift?)*

**21. What are the target wait times to see ward (incl. MAU) referrals (if any)?**

**22. Criteria by which your service sees outpatients, if at all:** (*If there is more than one pathway to being seen as an outpatient, please list them all and their criteria.)*

**23. Other activities your service undertakes, if any:** *(Many liaison services deliver teaching to staff, managers, students etc. Please describe all that happens and how frequently. Please also document any student attachments with your service. Please record any work undertaken by your service not captured above (eg. non-EDs 136s being undertaken). Last, if there is a service for frequent ED attenders, please describe it here, whether it is led by/delivered by liaison or not.)*

***Final queries:***

**24. Does your service use a competence framework? *If so, please give details.*** *(Particularly in how one is used. If no competence framework is actually in organisational use, but its existence is known and there are plans to use it, please document this too.)*

**25. Do you use FROM-LP outcome measures? If so, please indicate which elements:**

*IRAC*

*CGI-I*

*CORE-10*

*Patient Satisfaction Scale*

*Friends & Family Test*

*Referrer Satisfaction Scale*

**26. Do you use any other outcome measures? If so, please detail:**

**27. Is your service worse, similarly or better resourced than it was in July 2018?**

**28. What does your service do well?**

**29. What in your service is a challenge?** *(In particular, we would like to know if recruitment and retention is a challenge and what you may have done/are planning to meet that challenge.)*

**30. Is there anything else you would like to contribute to the survey?** *(Perhaps your service has piloted an initiative, or used winter monies creatively – please tell us the specifics of your unique service.)*

**Thank you for taking part in the Fifth Survey of Liaison Psychiatry in England (LPSE-5)**

**Please email your response to** [**lpse@exeter.ac.uk**](mailto:lpse@exeter.ac.uk)
