# Supplementary material for: Evaluating the provision of paediatric liaison psychiatry services in England
Source: BJPsych Open. 2023 Feb 1;9(2):e30. doi: 10.1192/bjo.2022.638 (PMC9970163; doi:10.1192/bjo.2022.638)
Supplement: Supplementary file 1 [file bjosup.zip › S205647242200638Xsup002.docx]

**Coding manual**

### Staffing:

- Paediatric staff do not count for this, adult (and older adult) workers only are included in this report.
- Only Band 5 or more count towards nursing (= MHP) numbers.
- Bands 5, 6, 7, 8 are equivalent.
- Where there are cradle to grave services, all the people are counted.
- Where services cover two or more hospitals, the people are divided equally, unless specified.
- Psychologists and pharmacists never count in MHP numbers. Other PAMs do, unless a specific boundaried role is described.
- Service manager ‘not specific to liaison’ is halved.
- People on sick leave are counted.
- ‘Part-time’ are assumed to be half time, if not specified.
- A fraction of a service leader is recorded, or a value of 0.2 FTE placed.
- Shared senior leadership is included. Distant reporting structures are not.
- Band 8s included if mentioned. If said to be not part of team, or off site, not counted.
- Where options are placed (“1 or 2 band 8s”) then we record the lower of the options.
- Where shifts covered by number of staff is specified, but the number of posts is not, a calculation assuming a full timer works 64% of days of the year for 8 hours was used to calculate the number of FTEs.
- Where FTE not specified, but hours are, a calculation based upon the hours worked was undertaken.
- Where there is another service which is melded with Liaison, the staffing is halved to assume equal activity in each.
- Drug and Alcohol workers of Band 5 or above count in the MHP numbers, regardless of which column they count in.
- Assistant psychologists are counted in ‘other’
- Personal assistants count in ‘other’.
- Therapists are counted in ‘other’.
- Administrative support is coded as 0.25 FTE if FTE not specified.
- Shared with other teams: FTE shared equally between the teams.
- Half clinical administrators are only counted toward MHPs total if they are Band 5 or above.
- ‘Tiny fragment of admin’ = 0.05 FTE
- Additional administrators: ‘psychology admins’ or ‘data analysts’ are included.
- Administrative support which is an unspecified part of a large bank are coded as 1 FTE.
- Consultants’ own personal assistants count in the administrators numbers.
- Drug and Alcohol workers are counted in the MHP numbers, regardless of source of funding, employer etc.
- Social workers working as MHPs are coded there. The minority with a boundaried social work role are coded in the ‘other’ column.

### ED Response Time Target:

- Services are included regardless of hours of operation.
- If 1 hour target for urgent or emergencies only, this counts.
- If intoxicated people can’t be seen, that does not undermine a 1 hour target.
- If the service is working towards a 1 hour target, this counts.
- Percentages within the target count.
